# CyKEGGParser: tailoring KEGG pathways to fit into systems biology analysis workflows

**DOI:** 10.12688/f1000research.4410.2

**Published:** 2014-08-14

**Authors:** Lilit Nersisyan, Ruben Samsonyan, Arsen Arakelyan

**Affiliations:** 1 Group of Bioinformatics, Institute of Molecular Biology, National Academy of Sciences of the Republic of Armenia, Yerevan, 0014, Armenia; 2 IUNETWORKS LLC, Yerevan, 0025, Armenia

## Abstract

The KEGG pathway database is a widely accepted source for biomolecular pathway maps. In this paper we present the CyKEGGParser app ( http://apps.cytoscape.org/apps/cykeggparser) for Cytoscape 3 that allows manipulation with KEGG pathway maps. Along with basic functionalities for pathway retrieval, visualization and export in KGML and BioPAX formats, the app provides unique features for computer-assisted adjustment of inconsistencies in KEGG pathway KGML files and generation of tissue- and protein-protein interaction specific pathways. We demonstrate that using biological context-specific KEGG pathways created with CyKEGGParser makes systems biology analysis more sensitive and appropriate compared to original pathways.

## Introduction

The KEGG pathway database is a widely accepted source for biomolecular pathway maps and has long been considered as the gold standard for pathway-based analyses due to well-formatted human-readable maps supplemented with machine-readable XML files (KGML), quality of curation and comprehensiveness ^1^. However, the KEGG pathway database suffers from a number of limitations that reduce the adaptability of the pathways for automated analysis. These include inconsistencies in KGML files supplied with each pathway image, such as absence of event or entity labels (e.g., links to other pathways or biological process labels), reversed directions for some associations, absence of some interactions, and inconsistent representation of compound interactions ^2^. Additionally, some features of KEGG pathways such as protein complex nodes and node duplication, enhance graphical representation, but reduce their machine-readability. Another limitation concerns abstractions (generalizations) used in pathway construction: (1) paralogous genes, not always occurring together in the same biological context, are grouped into single nodes, and (2) all the genes are assumed to be expressed and present in the same pathway. Additionally, the sources of information on interactions depicted in pathways differ in quality and the nature of interactions (indirect, physical, regulatory, etc.). Even accounting for these bottlenecks, the KEGG pathway database is still a highly valued resource, and we aimed to develop a tool that would make the best use of the information collected in it.

There is a wide variety of software that manipulate on KEGG pathways, both standalone and Cytoscape 3 apps, such as KEGGscape ( http://apps.cytoscape.org/apps/keggscape) for KEGG pathways visualization and data integration, and others. However, none of the available apps addresses inconsistencies in KGML files, and nor do they neither deal with abstractions of KEGG pathways. Herein, we describe CyKEGGParser app for Cytoscape 3 for KEGG pathway retrieval, visualization, adjustment for inconsistencies in computer-assisted manner, context-specific pathway generation, and exporting the pathways in KGML and BioPAX formats. CyKEGGParser is best suited for KEGG signaling pathways.

## Implementation

**Figure 1.**
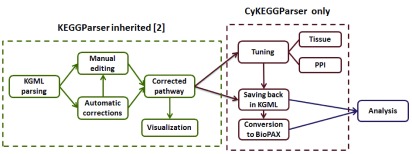
Graphical representation of CyKEGGParser use case.

The software is implemented in Java and is available as an app for Cytoscape 3. The general workflow of CyKEGGParser is presented in Figure 1.

### Pathway parsing and corrections

The input for parsing is KGML formatted files, either stored locally or downloaded from the web via REST-based KEGG API. The KEGG API can be used for individual downloads for academic use only; bulk download and non-academic usage requires a KEGG FTP subscription and license agreement ( http://www.kegg.jp/kegg/legal.html). The pathway selection dialogue provides a list of all KEGG pathways and organisms, however, if pathway KGML does not exist in the database the user will receive a warning message.

Each KGML file contains entries <pathway>, <entry> and <relation>, which are parsed using Java SAXParser API for reading XML files. The information contained in these entries is kept in Java objects which are instances of Graph, KeggNode and KeggRelation classes. These classes are implemented in CyKEGGParser and are independent of Cytoscape API. All the modifications applied by inconsistency correction algorithms are performed on these objects. Implementation of semi-automatic correction in CyKEGGParser is inherited from KEGGParser and described in detail by Arakelyan and Nersisyan ^2^.

Once the final Graph with its nodes and edges is created, it is converted into CyNetwork, CyNode and CyEdge objects using CyKEGGParser’s KeggNetworkCreator class. During the conversion, all the attributes contained in Graph, KeggNode and KeggEdge objects are set in respective Cytoscape attribute tables. More specifically, we use default CyTables for network, nodes and edges, populating them by creating a new CyColumn for each of the attributes and setting the values in CyRows during iteration over nodes and edges. After CyNetwork, CyNode and CyEdge objects are created, the algorithm iterates through each CyNode, creating a separate view for it and assigning coordinates from respective attributes for X and Y positions. Finally, CyKEGGParser creates attribute-based “kegg_vs” visual style, which is applied on the network with *VisualMappingManager* of Cytoscape API. However, any Cytoscape visual style may be applied depending on the user’s choice.

All the corrections performed on the network, as well as tuning and saving steps (described below) are tracked in separate log files (see the User Manual provided in the Cytoscape Help menu and at http://molbiol.sci.am/big/apps/cy_kp/jar/CyKEGGParser_User_Manual.pdf).

### Limitations for use of KEGG metabolic pathways

KEGG metabolic pathways, along with <relation/> entries, which characterize protein-protein interaction networks (enzyme interactions, in this case), also contain <reaction/> entries, characterizing compound interactions (chemical networks, http://www.kegg.jp/kegg/xml/docs/). Since CyKEGGParser relies on protein-protein interactions (PPI), parsing of metabolic pathways is not always as accurate as it is for signaling pathways. However, if only protein-protein interactions are of concern and if the KGML file contains respective <relation/> entries, CyKEGGParser will parse metabolic pathways similar to signaling ones.

### Pathway tuning

Along with the ability to modify the pathways by adding and deleting nodes and edges using Cytoscape-inherent tools, the user may as well customize (or “tune”) pathways according to specific biological context: particular tissue or cell type, and experimentally confirmed physical interactions.

***Tissue-specific tuning***. Tissue-specific tuning is aimed at providing the user with the ability to modify the networks based on genes expressed in a chosen cell/tissue type. Gene expression data for tuning is derived from BioGPS ( http://biogps.org/) experiments for human normal and cancer tissues, provided by GeneCards ( www.genecards.org), or may be supplied by the user (refer to User Manual for details). Along with specifying the source of data, the user chooses the tissue and specifies gene expression threshold.

The algorithm firstly clones the network preserving all the attributes, except for node and edge identifiers (those should be unique). Then it iterates over all the genes contained in the cloned network nodes, and removes the genes with expression values less than the specified threshold. If a node contains at least one gene that is expressed in current tissue, it remains in the network, otherwise it is removed. Nodes other than of type “gene” are preserved in the network.

***PPI based drill-down***. In KEGG pathways, node entries represent groups of paralogous genes that have similar functions or interaction profiles ^1^. The main incentive of PPI based pathway drill-down is to expand each node into its component genes and connect only those pairs of genes that have been shown to have true physical interactions. Together with tissue-specific tuning, this leads to generation of a “fine-tuned” network, in which all the components occur in the same biological context.

PPI data, retrieved from the String database ( http://string-db.org/), have been loaded in an internal MySQL database, located at the server of Bioinformatics Group of the Institute of Molecular Biology NAS RA ( http://molbiol.sci.am/big/). The user can choose the source of interactions from the list of databases (GRID, DIP, KEGG, MINT and PDB), as well as set interaction confidence score threshold, which is computed based on various evidence channels, adjusted for probability of randomly observing an interaction ^3^. The interactions are manually updated in the local My-SQL database and the version of String used is mentioned on the Tuning dialogue.

The algorithm initially creates a new network, copying all the nodes and node attributes from the former one. Afterwards, it drills down the new network through expanding each node of “gene” type into separate nodes for each member gene. Furthermore, the algorithm iterates over all the pairs of interacting nodes, and connects those members for which there is physical interaction in the corresponding PPI database. Attributes of newly assigned edges are copied from the former network table. After the drill down, duplicated nodes are combined into single ones, and isolated nodes are removed from the network.

### Saving

CyKEGGParser provides the functionality of saving the processed pathways back in valid KGML format, so that the modified pathways may be used outside of Cytoscape. All the modifications done to the network are saved in the attributes specific to KGML format. In addition, CyKEGGParser uses KEGGTranslator ^4^ binary file, embedded in the app package, for KGML conversion to BioPAX2 and BioPAX3 formats (see User Manual for details).

## Results and discussion

### Parsing and tuning of B Cell Receptor Signaling Pathway with CyKEGGParser

We have taken KEGG B Cell Receptor Signaling Pathway as an example to demonstrate CyKEGGParser functionality and its applicability in pathway-based biology research. B cells are important players in humoral immunity, and their main function is dependent on the B Cell Receptor Signaling Pathway, which is initiated by antigen binding to B cell receptor. We have tuned the B Cell Receptor Signaling Pathway based on BioGPS tissue-specific gene expression data in CD19 B and CD4 T cells (see *Implementation:, Pathway tuning* section), and compared pathway topologies in each case.

**Figure 2.**
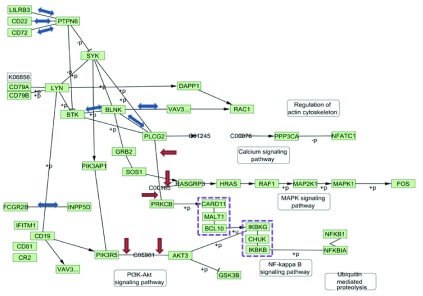
Visualization of KEGG B Cell Receptor Signaling Pathway after parsing and automatic correction. Red arrows indicate edges created by PCP corrections, blue double arrows indicate reversed interactions, and violet dashed rectangles indicate processed group nodes.

***Parsing and corrections***. Figure 2 shows the pathway parsed with CyKEGGParser with automatic correction options applied. These include three cases of protein-compound-protein (PCP) interaction processing, reversing binding interaction directions of seven edges and processing of two group nodes.

**Figure 3.**
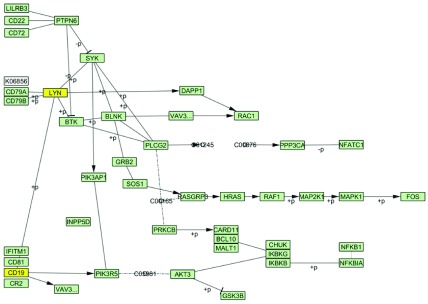
KEGG B cell signaling pathway tuned in CD19 B cells and CD4 T cells. Tuning was performed with 25 percentile threshold of gene expression values for each tissue. Highlighted in yellow are the nodes (“LYN” and “CD19”) not present in the pathway tuned in CD4 T cells.

***Tissue-specific tuning***. We performed B Cell Receptor Signaling Pathway tuning in CD19 B cells and CD4 T cells. Gene expression threshold was set to 25 percentile of gene expression values in the dataset. After tuning, from the 57 nodes available in the original pathway, 54 nodes remained in B cells and 52 nodes remained in T cells. Two nodes, namely, LYN, and CD19 are missing in the B Cell Receptor Signaling Pathway tuned in T cells ( Figure 3). Due to their topological importance in signal propagation from the receptors to the target nodes, absence of these two nodes leads to almost complete deactivation of the entire pathway in T cells.

**Figure 4.**
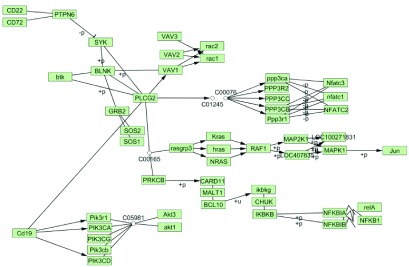
KEGG B cell signaling pathway after tissue specific and PPI-based tuning in CD19 B cells. Tissue-based tuning was performed with 25 percentile gene expression threshold. The confidence score for PPI-based tuning was set to 0.8 and all database sources were included.

***Protein-protein interaction based tuning***. The CD19 B cell tissue-specific version of the pathway was further tuned based on PPI. All the database sources (GRID, MINT, KEGG, DIP, PDB) were chosen and 0.8 confidence score threshold was set. Comparison of the PPI-tuned and the original networks showed that the node “VAV3…”, which contains three genes, VAV1, VAV2 and VAV3, was duplicated in the original pathway, but remained only in one place in the tuned network ( Figure 4). Moreover, of the three VAV member genes only VAV1 interacts with CD19 and BLNK, transducing the signal to rac1 and rac2 nodes. This observation is in accordance with a previously published study indicating VAV1 as the only player in B Cell Receptor Signaling Pathway ^5^.

### Effects of tissue-specific tuning on activity of cell signaling pathways

**Figure 5.**
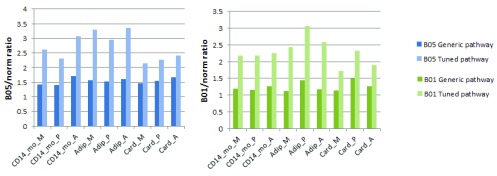
PSA score ratios of Calcium Signaling Pathway computed with simulated data. Target nodes are: M-“MAPK signaling pathway”, P-“Phosphatidylinositol signaling system”, A-“Apoptosis”. Tissues are: “CD14_mo”-CD14 monocytes, “Adip”-Adipocytes, “Card”-Cardiac myocytes.

To further demonstrate necessity of tissue-specific tuning for assessment of pathway activity changes, we compared pathway flows in original and tuned KEGG Calcium Signaling Pathways with three gene expression datasets (norm vs B05 and B01) in CD14 monocytes, Adipocytes, and Cardiac myocytes (see Supplementary Material for details). For calculations, we have used the Pathway Scoring Application for Cytoscape ^6^. The simulations show that pathway tuning increases the sensitivity of the pathway for signal flow analysis and thus the ability of the method to detect differentially expressed gene-related changes ( Figure 5).

Simulation Data Sets for CyKEGGParser **Dataset 1. PSA_scores_for_CalciumSignalingPathway.csv**. Description: Pathway scoring application scores for human Calcium signaling pathway, computed with gene expression data for CD14 Monocytes, Adipocytes and Cardiac myocytes with normal BioGPS gene expression data, and simulated B01 and B05 datasets. These data is presented in Figure 5 of the manuscript. **Dataset 2. CalciumSignalingPathway_gene_expression_data.csv**. Description: Gene expression data for genes belonging to KEGG Calcium signaling pathway from BioGPS experiments for normal human CD14 Monocytes, Adipocytes and Cardiac Mycocytes, and from two simulated datasets (B01 and B05). B05 and B01 datasets were generated from the normal tissue gene expression data, and by randomly assigning two-fold changes to genes based on Bernoulli distribution with probabilities 0.5 (B05) and 0.1 (B01), respectively.

## Conclusion

We have developed CyKEGGParser app for Cytoscape 3 that allows for import, correction, visualization, and tuning of KEGG pathways. Although KGML-based pathway import in Cytoscape has also been addressed by KGMLReader ( http://apps.cytoscape.org/apps/kgmlreader) and KEGGscape ( http://apps.cytoscape.org/apps/keggscape), semi-automatic correction and tuning-based enhancement of pathway specificity are unique and valuable features of CyKEGGParser. With this functionality we aim to maximize the effectiveness and sensitivity of gene expression-based systems biology analyses based on KEGG pathways.

## Software availability

App website: http://apps.cytoscape.org/apps/cykeggparser

Source code: https://github.com/lilit-nersisyan/cykeggparser

License: GNU Public License 3.0: https://www.gnu.org/licenses/lgpl.html

## Supplementary Material

### Gene expression data generation

We have analyzed KEGG Calcium Signaling Pathway with three gene expression datasets. As normal state (norm), we have taken BioGPS normal gene expression data for three tissues: CD14 monocytes, cardiac myocytes and adipocytes. For simulation of diseased states, we have taken the genes belonging to Calcium signaling pathway and randomly assigned two-fold change in a set of genes based on Bernoulli distribution with probabilities 0.5 (B05) and 0.1 (B01), respectively. In this way we have come up with one “diseased state” (B05) containing 50 and the other (B01) containing 8 differentially expressed genes (the data is provided in supplementary file “CalciumSignalingPathway_gene_expression_data.csv”).

Next we have tuned the pathway in CD14 monocytes, cardiac myocytes and adipocytes. For each cell type, the pathway was tuned with an arbitrary threshold of 6.5 corresponding to 27–33 percentiles of gene expression values in the three tissues.
