# Supplementary material for: CyKEGGParser: tailoring KEGG pathways to fit into systems biology analysis workflows
Source: F1000Res. 2014 Aug 14;3:145. Originally published 2014 Jul 1. [Version 2] doi: 10.12688/f1000research.4410.2 (PMC4215754; doi:10.12688/f1000research.4410.2)
Supplement: Peer review history

# Peer review: Referee response for version 2

**DOI:** 10.5256/f1000research.5389.r5832

**Published:** 2014-10-17

**Authors:** Augustin Luna

**Affiliations:** 1 Memorial Sloan-Kettering Cancer Center, New York, NY, USA

The authors have addressed my concerns and the information they provide will be helpful for future users. With respect to KEGG to BioPAX conversion, users of this Cytoscape plugin and make heavy use of the BioPAX output should now be aware from my comments here that the BioPAX export can be validated at the following URL:

http://biopax.baderlab.org/validator/check.html

in the case that they have any concerns of the validity of the output.

I have read this submission. I believe that I have an appropriate level of expertise to confirm that it is of an acceptable scientific standard.

# Peer review: Referee response for version 1

**DOI:** 10.5256/f1000research.4720.r5509

**Published:** 2014-08-06

**Authors:** Augustin Luna

**Affiliations:** 1 Memorial Sloan-Kettering Cancer Center, New York, NY, USA

The authors Lilit Nersisyan, Rouben Samsonyan and Arsen Arakelyan present the Cytoscape plugin/app, CyKEGGParser. The tool provides the functionality to parse KEGG KGML files and edit interactions based on expression data and external protein-protein interaction networks. This tool would be of interest to any researcher wishing to overlay experimental results onto curated pathways. 

**Issues:** 

**1:** Currently, the diagrams rendered in CyKEGGParser lack some elements of the original diagrams. Here is the link to the original " *B Cell Receptor Signaling Pathway*" 

http://www.genome.jp/kegg-bin/show_pathway?hsa04662

The original KEGG diagram contains vertical lines indicating transmembrane proteins that are missing from the CyKEGGParser version. There is also a missing interaction between RAC1 and " *Regulation of actin cytoskeleton*". Are both of the elements missing from KGML or are there limitations in the capabilities of Cytoscape to render elements, such as the vertical membrane lines? 

Is there any resource (by the authors or others) that keeps track issues with the KGML files, if this is the result of missing information in the KGML? 

**2:** In the PPI drill-down option, a confidence score threshold is provided, but it would be useful to direct readers to more information about this score and how to select appropriate values. 

What version of STRING do the authors use? Is this data automatically updated in their internal MySQL database? 

**3:** Currently, there seems to be a problem with the BioPAX export. I believe the problem is in the way KEGGTranslator is called in that lacks the --format option (I found the call to KEGGTranslator in the parsing.log file; this was tested on OS X 10.8.5). If this is indeed the problem, it should be easy to fix. It would be good if the authors validated the resulting BioPAX output from their KGML edits to see if any other issues arise. 

http://biopax.baderlab.org/validator/check.html

Missing ID cross-references (XRefs) is a likely error, but one that the authors might not be able to fix if this information is missing from the KGML file. 

**4:** "ppi" in " *Set ppi threshold*" should be capitalized in the Protein-protein interaction Settings window of the " *Pathway tuning settings*". 

There are some other minor issues in the dialog. Some of the panels do not seem large enough to accommodate the presented text. On the " *Gene Expression Settings*" with no file the text appears as " *No file selecte*"

CyKEGGParser seems stable and it performs the key function of helping users edit KEGG pathways, but it may require additional steps by users that want figures that mimic the aesthetics of the original KEGG diagrams (this may be unavoidable due to missing layout information in KGML and/or Cytoscape's inability to render all the KGML elements).

I have read this submission. I believe that I have an appropriate level of expertise to confirm that it is of an acceptable scientific standard, however I have significant reservations, as outlined above.

# Response

**Published:** 2014-08-07

**Authors:** Lilit Nersisyan

**Affiliations:** Institute of Molecular Biology NAS RA, Armenia

Thank you for reviewing our paper and your comments, the points you have mentioned are valuable for the app and comprehensiveness of the paper. Here is the detailed response and the description of changes we have made: 

**1:** **Currently, the diagrams rendered in CyKEGGParser lack some elements of the original diagrams. Here is the link to the original "B Cell Receptor Signaling Pathway"**

**http://www.genome.jp/kegg-bin/show_pathway?hsa04662**

**The original KEGG diagram contains vertical lines indicating transmembrane proteins that are missing from the CyKEGGParser version. There is also a missing interaction between RAC1 and "Regulation of actin cytoskeleton". Are both of the elements missing from KGML or are there limitations in the capabilities of Cytoscape to render elements, such as the vertical membrane lines?** 

**Is there any resource (by the authors or others) that keeps track issues with the KGML files, if this is the result of missing information in the KGML?** 

KEGG pathway map images, besides containing information about pathway nodes and their interactions, are also decorated with various graphical notations for visualization of spatial distribution of pathway components (i.e. cells, cell and nuclear membranes, organelles, and structural proteins). Since a KGML file solely includes nodes, relations between genes (gene products), compounds and maps (in some metabolic pathways only) and reactions, it is not possible to reconstruct any graphical decoration from a KGML file.

The same applies to present for protein – pathway and compound – pathway interactions, such is the case of *RAC1* and “Regulation of actin cytoskeleton”. In some cases, pathway map nodes are presented to indicate that some nodes and edges are a part of another pathway. In such cases, pathway nodes should not be linked to any node via interactions. In other cases, however, the link from a node to pathway node indicates functional relationship, which is not included in KGML files. In these cases, users can add these interactions manually and save them back in KGML format using CyKEGGParser’s functionality. 

Unfortunately, there is no resource that keeps track of the elements that are missing in KGML files, nor is it possible for the app to “guess” what is missing while parsing the actual KGML file.

**2:** **In the PPI drill-down option, a confidence score threshold is provided, but it would be useful to direct readers to more information about this score and how to select appropriate values.**

**What version of STRING do the authors use? Is this data automatically updated in their internal MySQL database?**

To clarify for the meaning of the confidence score, we have added the following paragraph in the User Manual and directed the user to String database’s help page for more details:

*“In String, the confidence score is derived by combining evidence about protein-protein interactions from various sources, adjusted for probability of randomly observing the interaction. More information about confidence score meanings and interaction sources can be found at the Help page of String database: http://string-db.org/newstring_cgi/show_info_page.pl?UserId=z7Cu7ePjQXnI&sessionId=rD_f8EsHGmAQ.”*

In the manuscript body, we have added the following paragraph, where we refer to the String database paper:

*“The user can choose the source of interactions from the list of databases (GRID, DIP, KEGG, MINT and PDB), as well as set interaction confidence score threshold, which is computed based on various evidence channels, adjusted for probability of randomly observing an interaction [3].”*

The local My-SQL database will be manually updated when new versions of String database are published. Currently, the String version 9.05 is used, and we are populating the database with interactions from version 9.1 at the moment. We have added a label on the tuning dialogue of the app, where the last updated version is seen, and added this information in the User Manual:

*“The interactions are manually updated in the local My-SQL database and the version of String used is mentioned on the Tuning dialogue.”*

**3:** **Currently, there seems to be a problem with the BioPAX export. I believe the problem is in the way KEGGTranslator is called in that lacks the --format option (I found the call to KEGGTranslator in the parsing.log file; this was tested on OS X 10.8.5). If this is indeed the problem, it should be easy to fix. It would be good if the authors validated the resulting BioPAX output from their KGML edits to see if any other issues arise.**

**http://biopax.baderlab.org/validator/check.html**

**Missing ID cross-references (XRefs) is a likely error, but one that the authors might not be able to fix if this information is missing from the KGML file.**

CyKEGGParser calls KEGGTranslator for BioPAX2 and BioPAX3 conversion, with --format BioPAX_level2 and --format BioPAX_level3 options applied, respectively. Could you, please, send us the log file, to see why the –– format option is missing?

KEGGTranslator performs a number of steps in order to retrieve the data missing in KGML files and come up with a valid BioPAX model, including completion of reactions, fixing invalid content of KGML entities and fetching cross-references, as described in http://www.biomedcentral.com/content/pdf/1752-0509-7-15.pdf. As In some cases, however, KEGGTranslator’s output is not successfully validated with BioPAX validator, mainly the BioPAX level 2 format. Since CyKEGGParser relies solely on command line calls to KEGGTranslator, there is nothing we can about these cases. However, we have tested whether the successfully validating outputs are also validated after pathway edits performed with CyKEGGParser or by the user in Cytoscape environment. Before calling KEGGTranslator, CyKEGGParser checks for absence of fields required by BioPAX2 and BioPAX3 formats, and adds default values as needed. This ensures that the edits do not induce problems for BioPAX conversion and further validation. The process of saving in KGML format and translating this format into BioPAX is described in the User Manual as follows:

*“KGML format saving assures that all the attributes required for BioPAX translation are available. For nodes, these are “entry: id” and “entry: type” attributes: these are assigned the default values (the next maximum id in the network and “gene” respectively). Node color and coordinates are not required for format conversion, however, if those are missing in the attributes, they will be assigned the values they have in Cytoscape. For interactions, the required attribute is the “type” attribute. If this attribute is missing in the network, it is assigned a value based on source and target node types, as follows: if both nodes are of type “gene” the interaction is assigned the type “PPrel”, if either of the nodes is of type “compound”, the type “PCrel” is assigned, otherwise the algorithm looks at the required format: in case of KGML and BioPAX_level2, it will assign “maplink” type to those interactions, where one of the nodes is of type “Map”, and will give the default value “PPrel” otherwise; in case of BioPAX_level3 the interaction will be removed from the network, since the latter format does not allow for interactions between non-physical entities.”*

**4: "ppi" in "Set ppi threshold" should be capitalized in the Protein-protein interaction Settings window of the "Pathway tuning settings".**

**There are some other minor issues in the dialog. Some of the panels do not seem large enough to accommodate the presented text. On the "Gene Expression Settings" with no file the text appears as "No file selecte".**

We have changed “ppi” to “PPI” in the “Set ppi threshold” of the dialogue, and added more space to file selection label in the “Gene Expression Settings” tab.

# Peer review: Referee response for version 1

**DOI:** 10.5256/f1000research.4720.r5481

**Published:** 2014-07-18

**Authors:** Hans Binder

**Affiliations:** 1 Interdisciplinary Centre for Bioinformatics, University of Leipzig, Leipzig, Germany

The article *CyKEGGParser: tailoring KEGG pathways to fit into systems biology analysis workflows *by Lilit Nersisyan, Rouben Samsonyan and Arsen Arakelyan presents a comprehensive software tool allowing to edit KEGG pathways based on additional information taken, e.g., from tissue expression data and/or protein interaction networks. The functionalities are well described and illustrated using selected pathways in the context of B-cell signalling.

In my opinion, the impact of the contribution is high especially for researchers who are interested in different kinds of biological networks and particularly who aim at interpreting experimental results in terms pathway activities.

I have read this submission. I believe that I have an appropriate level of expertise to confirm that it is of an acceptable scientific standard.
